# Development of an Immune Infiltration-Related Eight-Gene Prognostic Signature in Colorectal Cancer Microenvironment

**DOI:** 10.1155/2020/2719739

**Published:** 2020-08-27

**Authors:** Beilei Wu, Lijun Tao, Daqing Yang, Wei Li, Hongbo Xu, Qianggui He

**Affiliations:** ^1^Medical Examination Center, Wenzhou Central Hospital, The Dingli Clinical College of Wenzhou Medical University, Wenzhou, 325000 Zhejiang, China; ^2^Department of Traumatic Surgery, The First Affiliated Hospital of Wenzhou Medical University, Wenzhou, 325000 Zhejiang, China; ^3^Department of Colorectal Surgery, Wenzhou Central Hospital, The Dingli Clinical College of Wenzhou Medical University, Wenzhou, 325000 Zhejiang, China

## Abstract

**Objective:**

Stromal cells and immune cells have important clinical significance in the microenvironment of colorectal cancer (CRC). This study is aimed at developing a CRC gene signature on the basis of stromal and immune scores.

**Methods:**

A cohort of CRC patients (*n* = 433) were adopted from The Cancer Genome Atlas (TCGA) database. Stromal/immune scores were calculated by the ESTIMATE algorithm. Correlation between prognosis/clinical characteristics and stromal/immune scores was assessed. Differentially expressed stromal and immune genes were identified. Their potential functions were annotated by functional enrichment analysis. Cox regression analysis was used to develop an eight-gene risk score model. Its predictive efficacies for 3 years, 5 years, overall survival (OS), and progression-free survival interval (PFI) were evaluated using time-dependent receiver operating characteristic (ROC) curves. The correlation between the risk score and the infiltering levels of six immune cells was analyzed using TIMER. The risk score was validated using an independent dataset.

**Results:**

Immune score was in a significant association with prognosis and clinical characteristics of CRC. 736 upregulated and two downregulated stromal and immune genes were identified, which were mainly enriched into immune-related biological processes and pathways. An-eight gene prognostic risk score model was conducted, consisting of CCL22, CD36, CPA3, CPT1C, KCNE4, NFATC1, RASGRP2, and SLC2A3. High risk score indicated a poor prognosis of patients. The area under the ROC curves (AUC) s of the model for 3 years, 5 years, OS, and PFI were 0.71, 0.70, 0.73, and 0.66, respectively. Thus, the model possessed well performance for prediction of patients' prognosis, which was confirmed by an external dataset. Moreover, the risk score was significantly correlated with immune cell infiltration.

**Conclusion:**

Our study conducted an immune-related prognostic risk score model, which could provide novel targets for immunotherapy of CRC.

## 1. Introduction

CRC, as a heterogeneous disease, is a common cause of cancer-related deaths worldwide [1]. TNM staging is usually considered to be one of the main tools for CRC prognosis [2]. However, the prognosis varies greatly among CRC patients with the same TNM stage, suggesting that the current TNM stage does not well provide complete prognostic information for CRC patients. Therefore, it is necessary to adopt a new strategy to increase the predictive efficiency of prognosis and survival outcomes of CRC patients.

Due to the considerable heterogeneity between CRCs, determination of the optimal treatment strategy at the individual level faces the large challenges. Thus, it is an urgent need to conduct robust models to identify high-risk CRC patients and to find novel molecular targets. In the tumor microenvironment (TME), stromal and immune cells are involved in the development of CRC [3, 4]. Increasing evidence suggests that stromal and immune cells possess critical clinical significance for CRC. It has been reported that stromal cells can contribute to transcriptome and clinical features of CRC subtype [5]. Furthermore, stromal gene expression can more robustly predict the prognosis of CRC subtypes compared to epithelial tumor cells [6]. In a large cohort of CRC patients, infiltrating immune cell data could better predict patients' survival than histopathological methods [7]. Growing studies have found that infiltrating immune cells are involved in chemoresistance [8] and metastasis [9]. Thus, it is essential to further analyze the biological characteristics of stromal and immune genes and to determine their prognostic value for CRC patients. However, there is a lack of stromal and immune scores that can predict CRC patients' prognosis based on multiple clinical factors. Moreover, robust prognostic models on the basis of stromal and immune scores are also lacking.

In this study, we established a reliable prognostic immune-related risk score for CRC. Our results could offer novel insights for prediction of CRC patients' prognosis and development of individualized immunity therapy strategies.

## 2. Materials and Methods

### 2.1. CRC Datasets

**Figure 1:**
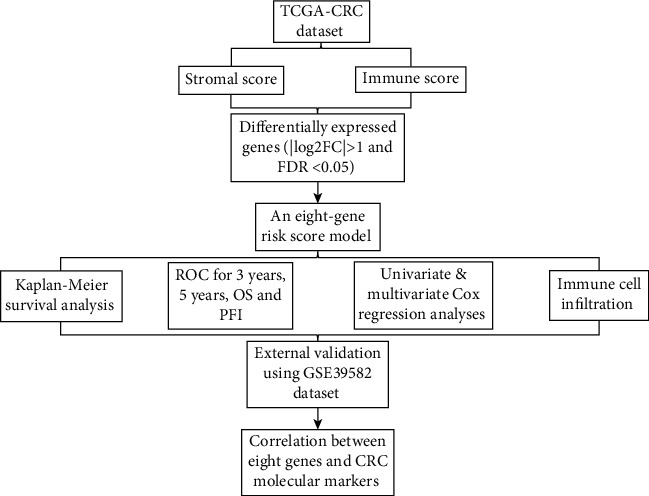
An overview of the workflow.

TCGA RNA-seq data (including Counts and FPKM data) of GDC CRC (including 469 CRC tissue samples and 41 adjacent normal tissue samples) were downloaded from the xenabrowser website (https://xenabrowser.net/). Among all CRC samples, 433 samples contained complete clinical information, including gender, age, TNM stage, tumor grade, microsatellite instability (MSI), and mismatch repair (MMR). The clinical information of 433 CRC patients is listed in Table 1. Survival information including OS status, OS time, progression-free survival (PFS) status, and PFS time was derived from the pan-cancer on the GDC website (https://gdc.cancer.gov/about-data/publications/PanCan-Clinical-2018). Furthermore, mutation data (including BRAF, KRAS, and TP53) were from CRC MuTect. An overview of the workflow is shown in Figure 1.

### 2.2. Estimation of Stromal/Immune Scores

ESTIMATE algorithm was used to calculate the stromal/immune scores on the basis of unique expression profiles of stromal/immune cells by the ESTIMATE package in R (https://bioinformatics.mdanderson.org/estimate/) [10].

### 2.3. Kaplan-Meier Survival Analysis

According to the optimal cutoff of stromal/immune scores, CRC samples were classified into high and low stromal/immune score groups. Kaplan-Meier plot of overall survival between the two groups was analyzed, and the results were evaluated by log-rank test.

### 2.4. Correlation between Clinical Characteristics and Stromal/Immune Scores

To probe into the clinical significance of stromal/immune scores, we analyzed the correlation between clinical characteristics (including pathologic T, pathologic N, pathologic M, and tumor stage) and stromal/immune scores.

### 2.5. Differential Expression Analysis

Differential expression analysis between high and low stromal/immune score groups was carried out using the edgeR package in R, following the screening criteria of ∣log2 fold change (FC) | >1 and FDR (adjusted p value) < 0.05. Then, up- or downregulated stromal/immune genes were intersected by the VennDiagram package in R, respectively.

### 2.6. Functional Enrichment Analysis

Gene Ontology (GO) and Kyoto Encyclopedia of Genes and Genomes (KEGG) pathway enrichment analyses of differentially expressed stromal and immune genes were carried out through the clusterProfiler package in R [11]. GO analysis contains three terms, cellular component (CC), molecular function (MF), and biological process (BP). *p* value after adjustment < 0.05 was significantly enriched.

### 2.7. Protein-Protein Interaction (PPI) Analysis

PPI analyses of differentially expressed stromal and immune genes were carried out via The Search Tool for the Retrieval of Interacting Genes (STRING, https://string-db.org/; version 11) [12]. Then, the PPI network was visualized through Cytoscape (version 3.7.2) [13].

### 2.8. Univariate and Multivariate Cox Regression Analyses

Univariate cox regression analysis of differentially expressed stromal and immune genes was carried out via the survival package in R. Genes with *p* value < 0.05 were screened for multivariate cox regression analysis. To validate the sensitivity and accuracy of the risk score for prediction of CRC, an ROC curve was drawn to evaluate the predictive performance of the risk core for 3 years, 5 years, OS, and PFI using the “tdROC” package in R. The results were visualized with the “ggplot2” package in R. The AUC was then calculated. The GSE39582 dataset from the Gene Expression Omnibus (GEO) database (https://www.ncbi.nlm.nih.gov) was used to validate the prognostic value of the risk score. The dataset was composed of 566 CRC samples.

### 2.9. Immune Infiltration Analysis

The tumor-immune infiltration cells including B cells, CD4+T cells, CD8+T cells, macrophages, neutrophils, and dendritic cells were estimated via the TIMER (https://cistrome.shinyapps.io/timer/) [14]. Spearman's correlation between the risk score and the infiltrating levels of immune cells was evaluated through the psych package in R. Furthermore, we also assessed the correlation between the genes in the risk score and marker genes of immune cells. The strength of correlation followed the criteria: 0.7 ≤ ∣ *r*  | ≥1 suggested a high correlation, 0.3 ≤ ∣ *r*  | <0.7 suggested a moderate correlation, and 0 < ∣ *r*  | <0.3 suggested a weak correlation [15].

## 3. Results

### 3.1. Immune Score Is in Significant Association with Prognosis and Clinical Features of CRC Patients

**Figure 2:**
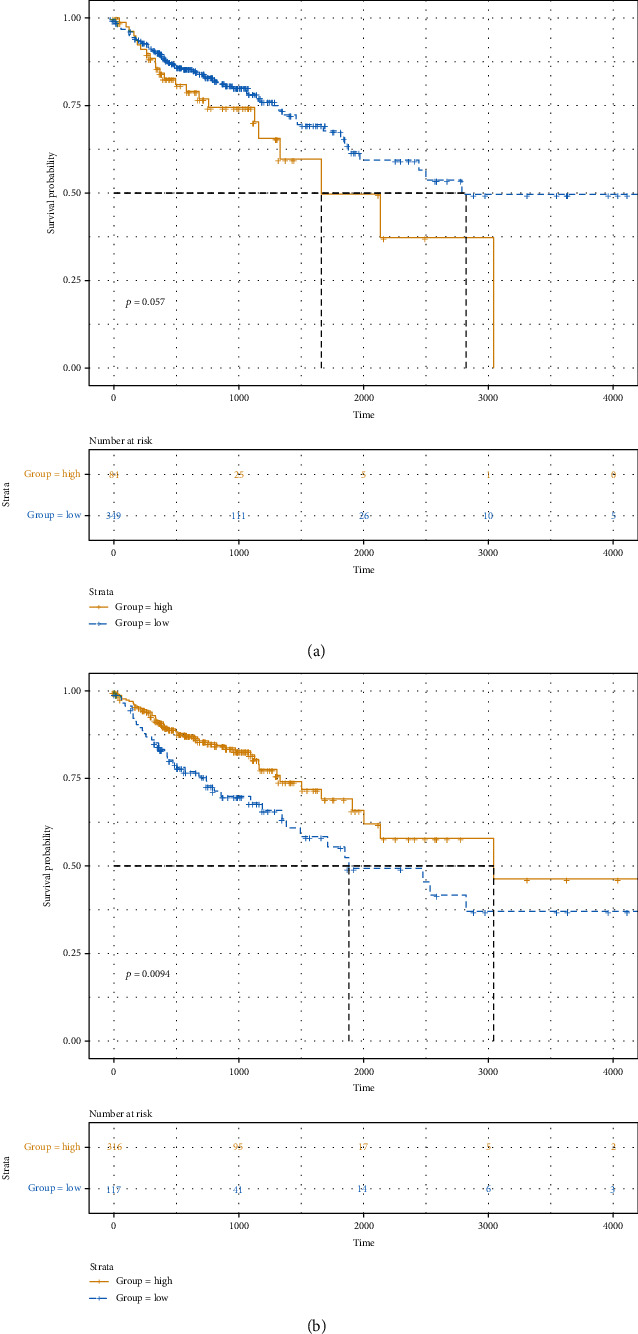
The correlation between stromal/immune scores and CRC patients' survival outcomes. (a) Stromal score. (b) Immune score. The *x*-axis suggests overall survival time and *y*-axis represents survival probability.

**Figure 3:**
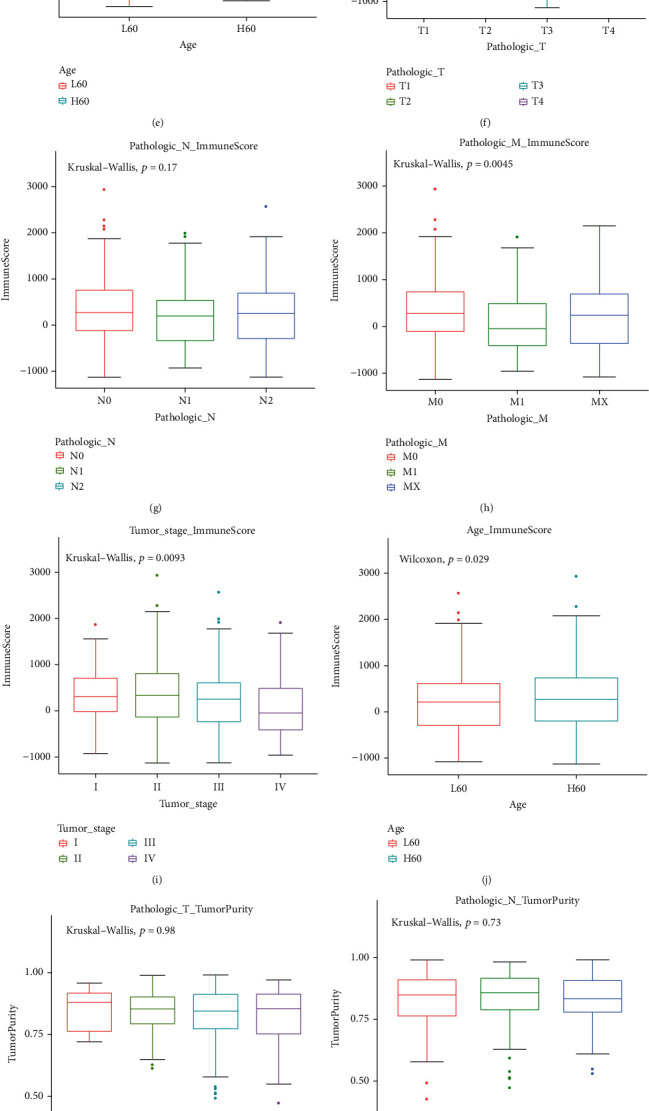
The correlation between stromal/immune/ESTIMATE scores and CRC patients' clinical features. Stromal score is not associated with (a) pathologic T (*p* value = 0.61), (b) pathologic N (*p* value = 0.28), (c) pathologic M (*p* value = 0.63), (d) tumor stage (*p* value = 0.68), and (e) age (*p* value = 0.76). Immune score is not correlated with (f) pathologic T (*p* value = 0.88) and (g) pathologic N (*p* value = 0.17). Immune score significantly associated with (h) pathologic M (*p* value = 0.0045) and (i) tumor stage (*p* value = 0.0093). Immune score is not correlated with (j) age (*p* value = 0.29). ESTIMATE scores are not correlated with (k) pathologic T (*p* value = 0.98), (l) pathologic N (*p* value = 0.73), (m) pathologic M (*p* value = 0.095), (n) tumor stage (*p* value = 0.28), and (o) age (*p* value = 0.74).

According to the optimal cutoff of stromal/immune scores, the CRC patients were divided into two groups. Kaplan-Meier OS analysis results showed that patients with high stromal score had shorter OS time than those with low stromal score; however, it was not statistically significant (Figure 2(a); *p* value = 0.057). As depicted in Figure 2(b), we found that patients with low immune score implied a poor prognosis (*p* value = 0.0094). Furthermore, we analyzed the correlation between stromal/immune scores and clinical features. As depicted in the results, stromal score was not significantly associated with pathologic T (Figure 3(a); *p* value = 0.61), pathologic N (Figure 3(b); *p* value = 0.28), pathologic M (Figure 3(c); *p* value = 0.63), tumor stage (Figure 3(d); *p* value = 0.68), and age (Figure 3(e); *p* value = 0.76). Similarly, we also found that there was no statistical significance between immune score and pathologic T (Figure 3(f); *p* value = 0.88) and pathologic N (Figure 3(g); *p* value = 0.17). As expected, immune score was in significant association with pathologic M (Figure 3(h); *p* value = 0.0045) and tumor stage (Figure 3(i); *p* value = 0.0093). However, no significant correlation between immune score and age was found in Figure 3(j) (*p* value = 0.29). Furthermore, ESTIMATE scores were not correlated with pathologic T (Figure 3(k); *p* value = 0.98), pathologic N (Figure 3(l); *p* value = 0.73), pathologic M (Figure 3(m); *p* value = 0.095), tumor stage (Figure 3(n); *p* value = 0.28), and age (Figure 3(o); *p* value = 0.74). These findings indicated that immune score was in significant association with CRC patients' prognosis and clinical features.

### 3.2. Identification of Differentially Expressed Stromal and Immune Genes for CRC

**Figure 4:**
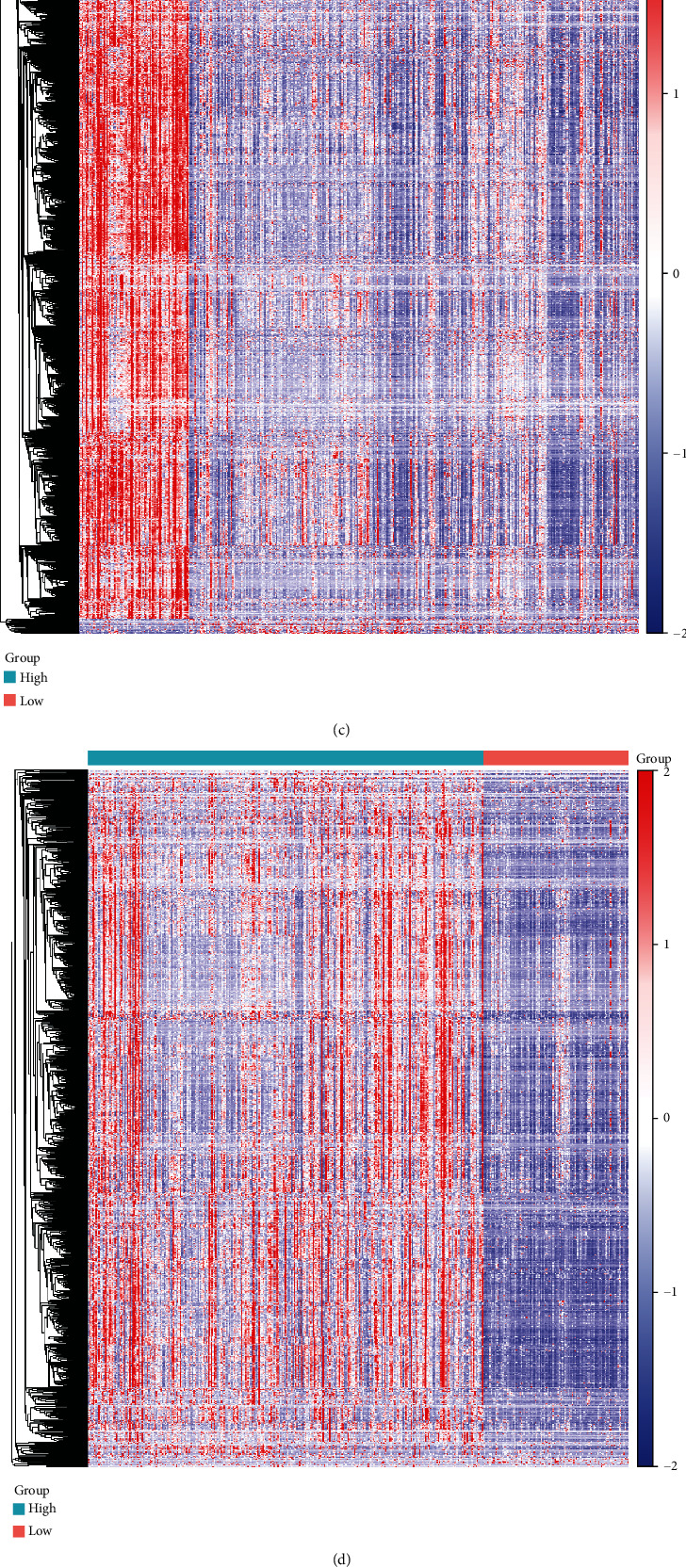
Identification of differentially expressed stromal and immune genes for CRC. Volcano plot showing up- (red) and downregulated (blue) stromal/immune genes in the high stromal score group (a) and immune score group (b). Heat maps depicting all DEGs in the high/low stromal score group (c) and high/low immune score group (d). Venn diagram showing common upregulated (e) and downregulated (f) stromal and immune genes.

**Figure 5:**
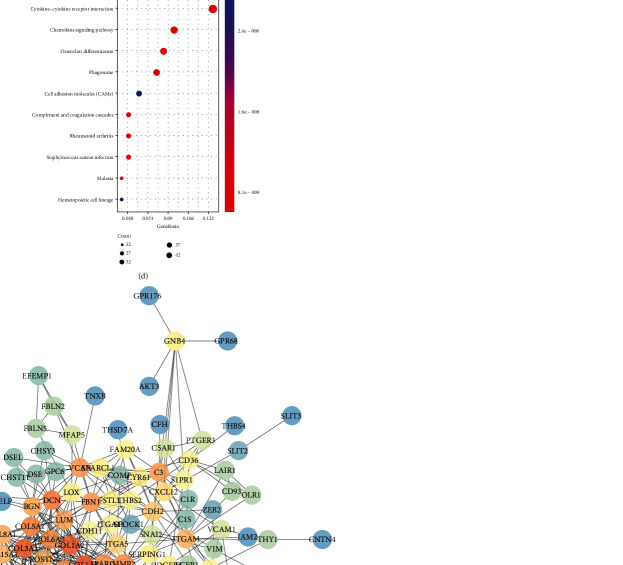
Functional enrichment and PPI analyses of common stromal and immune DEGs. (a) The top the BP terms of GO enrichment analysis. (b) The top ten CC terms of GO enrichment analysis. (c) The top ten MF terms of GO enrichment analysis. (d) The top ten KEGG pathway analysis. (e) PPI network construction.

We analyzed differentially expressed genes (DEGs) with ∣log2FC | >1 and FDR < 0.05 between the high and low stromal/immune score groups. As volcano plots, there were 1197 up- and 28 downregulated stromal genes in the high stromal score group (Figure 4(a)). Furthermore, 899 immune genes were upregulated and eight immune genes were downregulated in the high immune score group (Figure 4(b)). Hierarchical clustering analysis results showed that both stromal DEGs and immune DEGs could distinguish high stromal/immune score from low stromal/immune score (Figures 4(c) and 4(d)). 736 genes were upregulated both in high stromal and immune scores (Figure 4(e)). Moreover, among eight downregulated immune genes, two genes were also downregulated in the high stromal score group (Figure 4(f)). We further performed functional enrichment analysis of these common stromal and immune genes. These genes were significantly correlated with inflammatory biological processes like regulation of inflammatory response and pathways such as cytokine-cytokine receptor interaction and chemokine signaling pathway (Figures 5(a)–5(d)). As shown in the PPI network, COL6A2, COL6A1, COL5A2, C1S, and C1R were the top five genes, which were considered hub genes (Figure 5(e)).

### 3.3. Construction of an Eight-Gene Prognostic Signature for CRC

**Figure 6:**
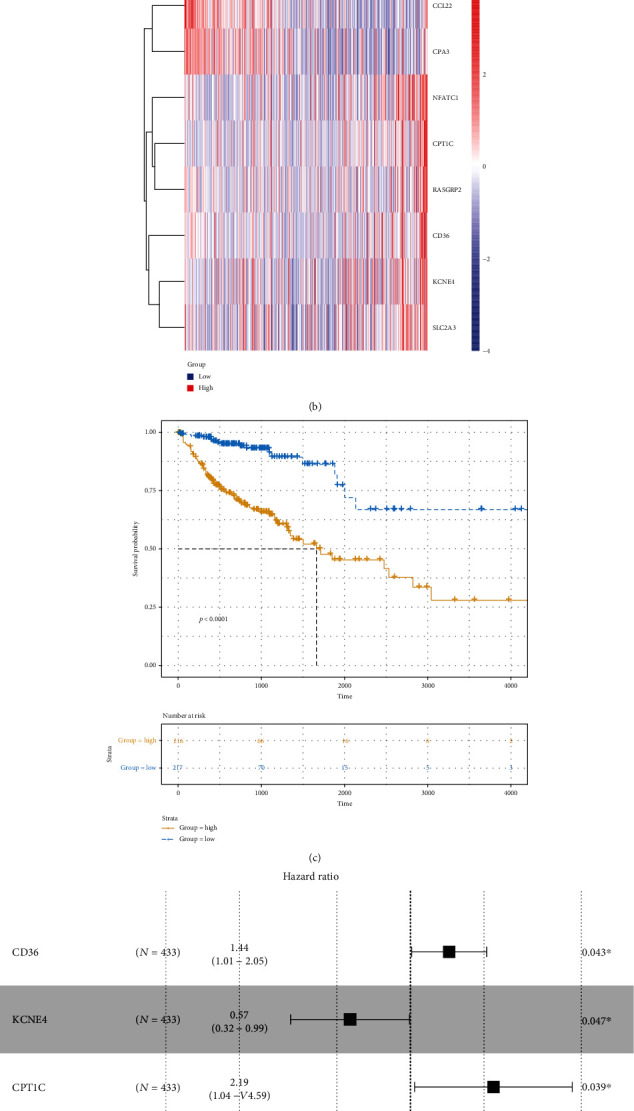
Construction of an eight-gene prognostic signature for CRC. (a) Risk score distribution and survival status. (b) Heat maps showing the expression patterns of the eight genes between high and low risk score. (c) Kaplan-Meier survival analysis of the model. (d) Forest plot of the eight genes for CRC. (e, f) ROC curve of the model for 3-year, 5-year, OS, and PFI.

Among 738 differentially expressed stromal and immune genes, 23 genes were significantly associated with CRC patients' prognosis according to univariate Cox regression analysis results. Of them, 20 genes were risk factors, and the remaining three (CCL22, CPA3, and MMP1) were protective factors (Table 2). These genes were used for multivariate Cox regression analysis. Finally, an eight-gene signature was constructed for CRC. The risk score was calculated on the basis of the coefficients and expression values of the eight genes. All CRC patients were divided into two groups in accordance with the median value of risk score (Figure 6(a)). Heat maps depicted the difference in expression patterns of the eight genes (including CD36, KCNE4, CPT1C, SLC2A3, RASGRP2, NFATC1, CCL22, and CPA3) between the high and low risk scores (Figure 6(b)). As shown in Figure 6(c), the risk score was capable of predicting CRC patients' prognosis. High risk score implied a poor prognosis (*p* value < 0.0001). Among the eight genes, KCNE4 and CCL22 were protective factors of CRC, while CD36, CPT1C, SLC2A3, RASGRP2, NFATC1, and CPA3 were risk factors of CRC, as shown in the forest diagram (Figure 6(d)). We further validated the sensitivity and accuracy of the model. AUCs of the model for 3 years, 5 years, OS, and PFI were 0.71, 0.70, 0.73, and 0.66, respectively (Figures 6(e) and 6(f)). Thus, the risk score model could well predict CRC patients' prognosis, with high sensitivity and accuracy. As shown in the multivariate Cox regression analysis, the model and MMR could become independent factors for CRC prognosis after adjustment of other clinical characteristics (Table 3).

### 3.4. Eight Genes in the Risk Score Model Are Significantly Associated with CRC Patients' Prognosis

**Figure 7:**
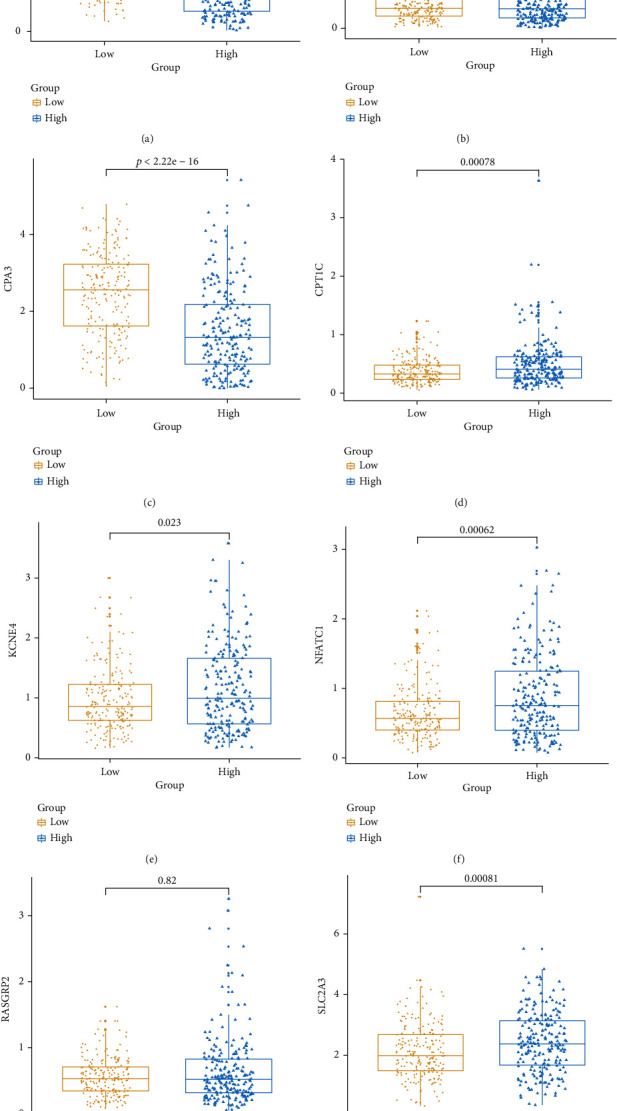
Box plots depicting the expression patterns of the eight genes in the risk score model between high and low risk score. (a) CCL22. (b) CD36. (c) CPA3. (d) CPT1C. (e) KCNE4. (f) NFATC1. (g) RASGRP2. (h) SLC2A3.

**Figure 8:**
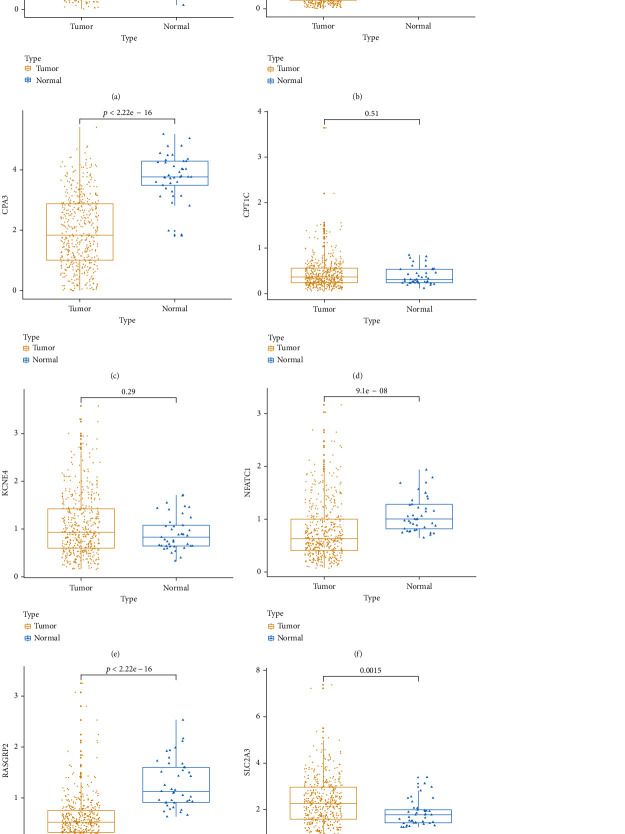
Box plots showing the expression patterns of the eight genes in the risk score model between CRC samples and normal samples. (a) CCL22. (b) CD36. (c) CPA3. (d) CPT1C. (e) KCNE4. (f) NFATC1. (g) RASGRP2. (h) SLC2A3.

**Figure 9:**
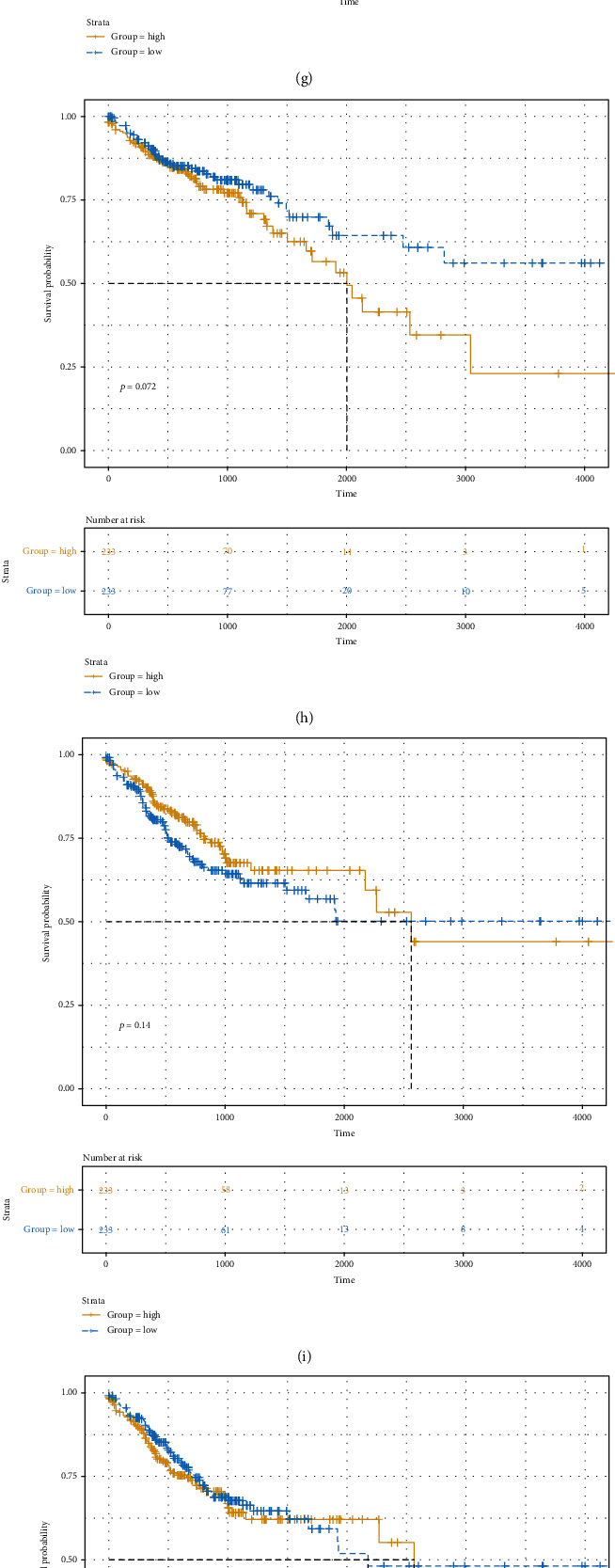
Kaplan-Meier plot of OS and PFS for the eight genes in the risk score model between high and low risk score. Kaplan-Meier OS results for (a) CCL22, (b) CD36, (c) CPA3, (d) CPT1C, (e) KCNE4, (f) NFATC1, (g) RASGRP2, and (h) SLC2A3. Kaplan-Meier PFS results for (i) CCL22, (j) CD36, (k) CPA3, (l) CPT1C, (m) KCNE4, (n) NFATC1, (o) RASGRP2, and (p) SLC2A3.

Box plot depicted the difference in expression patterns of CCL22 (Figure 7(a)), CD36 (Figure 7(b)), CPA3 (Figure 7(c)), CPT1C (Figure 7(d)), KCNE4 (Figure 7(e)), NFATC1 (Figure 7(f)), RASGRP2 (Figure 7(g)), and SLC2A3 (Figure 7(h)) between the high risk score and low risk score. Among them, CCL22 (*p* value < 2.22*e*-16), CPA3 (*p* value < 2.22*e*-16), CPT1C (*p* value = 0.00078), KCNE4 (*p* value = 0.023), NFATC1 (*p* value = 0.00062), and SLC2A3 (*p* value = 0.00081) were differentially expressed between the high and low risk scores. Furthermore, the expression levels of these genes between CRC samples and normal samples were visualized (Figures 8(a)–8(g)). CD36 (*p* value < 2.22*e*-16), CPA3 (*p* value < 2.22*e*-16), NFATC1 (*p* value = 9.1*e*-08), and RASGRP2 (*p* value < 2.22*e*-16) were highly expressed and SLC2A3 (*p* value = 0.0015) was lowly expressed in tumor samples. As shown in Figures 9(a)–9(h), low expression of CCL22 (*p* value = 0.0047) and CPA3 (*p* value = 0.035) indicated shorter OS time than high expression. Moreover, we found that highly expressed CPT1C (*p* value = 0.0017), KCNE4 (*p* value = 0.002), and SLC2A3 (*p* value = 0.0048) was significantly correlated with poor PFS (Figures 9(i)–9(p)).

### 3.5. The Eight-Gene Model Is in Significant Correlation with Immune Cell Infiltration

**Figure 10:**
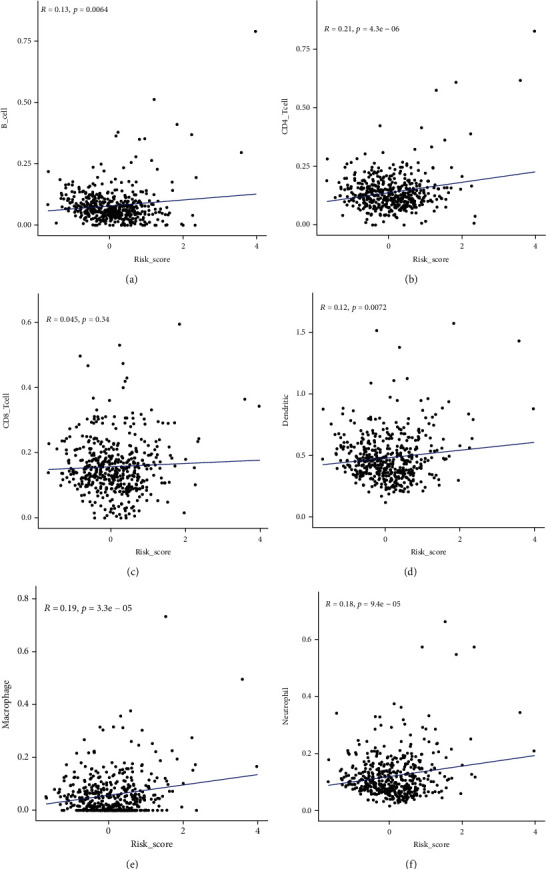
The correlation between the eight-gene risk score and immune cell levels. (a) B cell. (b) CD4+T cell. (c) CD8+T cell. (d) Dendritic cell. (e) Macrophage. (f) Neutrophil.

**Figure 11:**
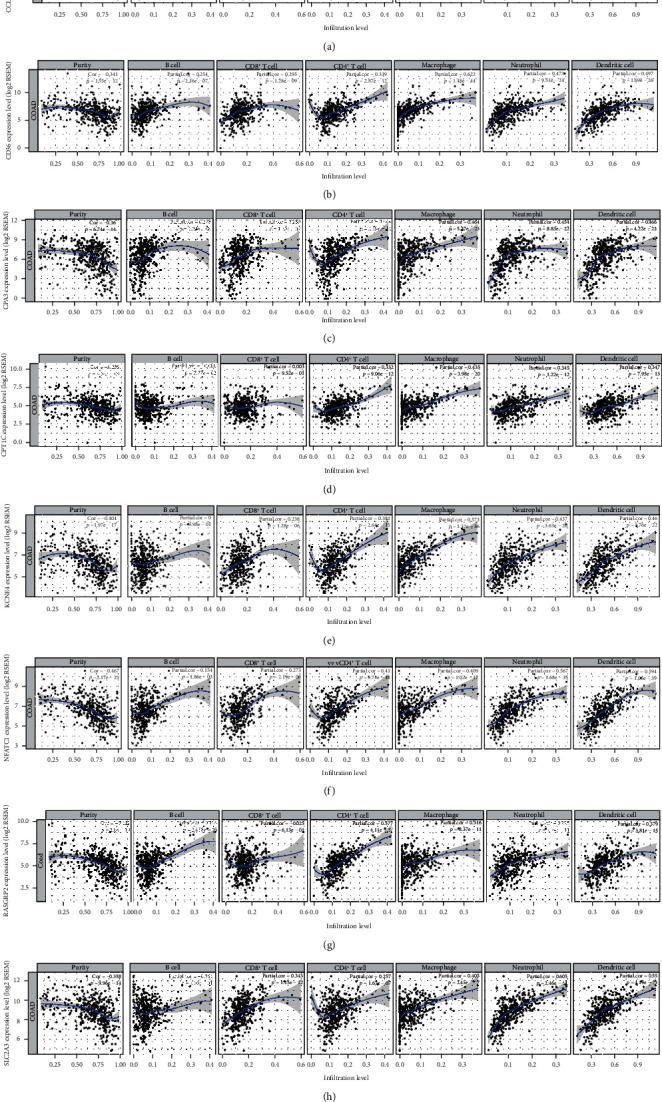
The correlation between the eight genes in the risk score model and immune cell levels. (a) CCL22. (b) CD36. (c) CPA3. (d) CPT1C. (e) KCNE4. (f) NFATC1. (g) RASGRP2. (h) SLC2A3.

The correlation between the model and the infiltrating levels of six immune cells was analyzed. We found that the model was in significant association with the infiltrating levels of six immune cells, including B cell (Figure 10(a); *R* = 0.13, *p* value = 0.0064) and CD4+T cell (Figure 10(b); *R* = 0.21, *p* value =4.3*e*-06). However, no significant correlation between the model and CD8+T cell was found in Figure 10(c) (*R* = 0.045, *p* value = 0.34). Furthermore, there were distinct correlations between the model and dendritic cell (Figure 10(d); *R* = 0.12, *p* value = 0.0072), macrophage (Figure 10(e); *R* = 0.19, *p* value = 3.3*e*-05), neutrophil (Figure 10(f); *R* = 0.18, *p* value = 9.4*e*-05). We also found that the expression levels of the eight genes in the model were significantly correlated with the infiltrating levels of six immune cells, including CCL22 (Figure 11(a)), CD36 (Figure 11(b)), CPA3 (Figure 11(c)), CPT1C (Figure 11(d)), KCNE4 (Figure 11(e)), NFATC1 (Figure 11(f)), RASGRP2 (Figure 11(g)), and SLC2A3 (Figure 11(h)). Moreover, the eight genes were in significant association with markers of immune cells (Supplementary Table 1). These results suggested that the model could be in association with immune cell infiltration.

### 3.6. Validation of the Risk Score Using an Independent Dataset

**Figure 12:**
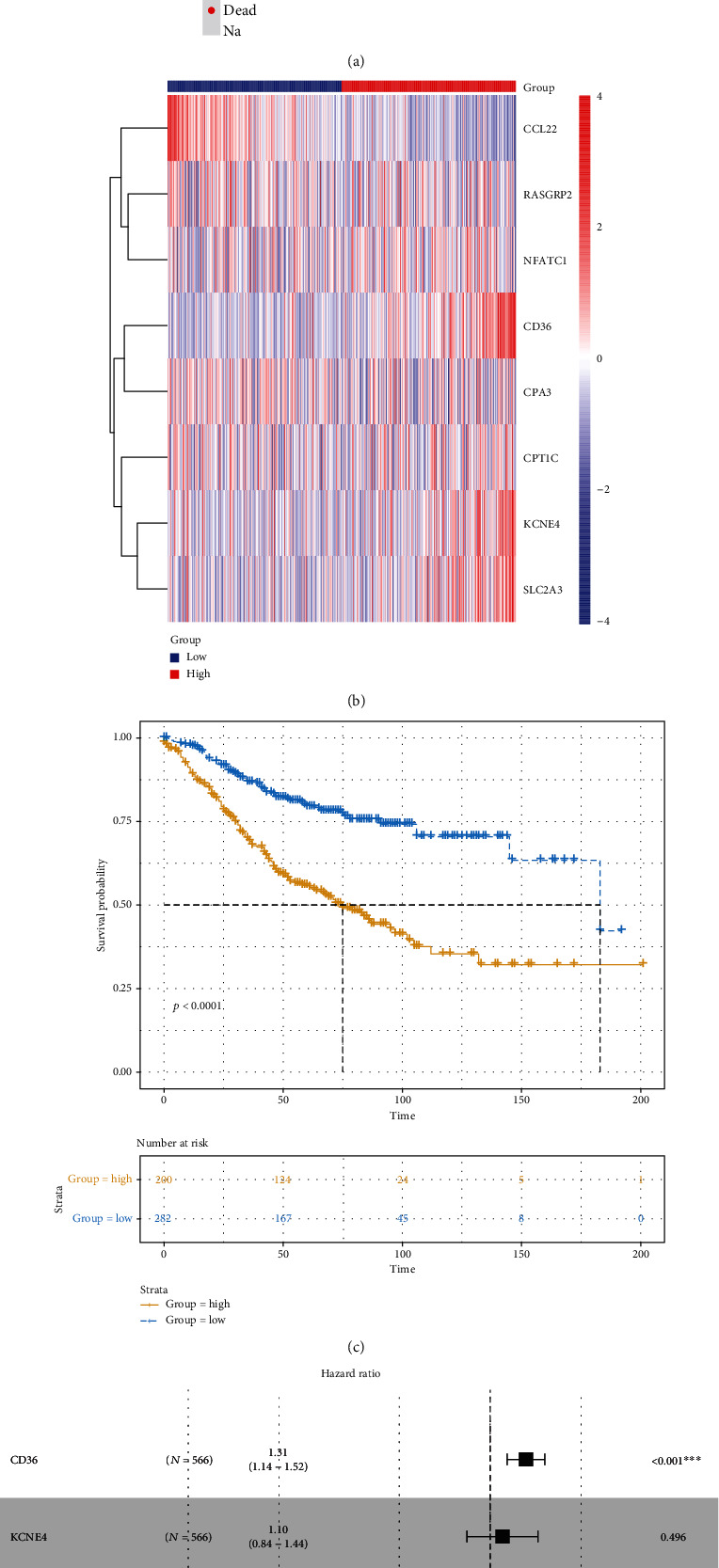
Validation of the eight-gene prognostic signature for CRC using an independent dataset. (a) Risk score distribution and survival status. (b) Heat maps showing the expression patterns of the eight genes between high- and low risk score. (c) Kaplan-Meier survival analysis of the model. (d) Forest plot of the eight genes for CRC. (e) ROC curve of the model for 3 years and 5 years.

**Figure 13:**
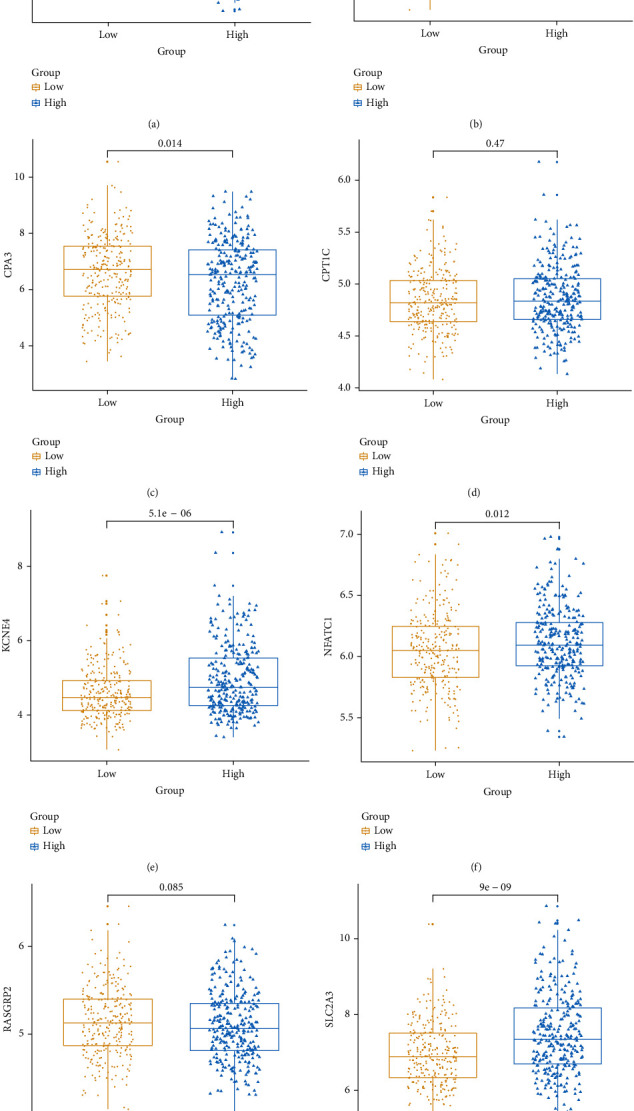
The expression patterns of the eight genes in the risk score model between high and low risk score were validated using an independent dataset. (a) CCL22. (b) CD36. (c) CPA3. (d) CPT1C. (e) KCNE4. (f) NFATC1. (g) RASGRP2. (h) SLC2A3.

Based on 566 CRC samples from the GSE39582 dataset, the prognostic value of the risk score was validated. The risk score distribution and survival status of CRC patients are shown in Figure 12(a). Heat maps showed the expression differences of CD36, KCNE4, CPT1C, SLC2A3, RASGRP2, NFATC1, CCL22, and CPA3 between the high and low risk scores (Figure 12(b)). As expected, CRC patients with high risk score had a poorer prognosis than those with low risk score (Figure 12(c)). Among the eight genes, CD36, NFATC1, and CCL22 were significantly associated with prognosis of CRC patients (Figure 12(d)). AUCs of the model for 3 years and 5 years were 0.65 and 0.66, respectively (Figure 12(e)), indicating that the risk score could well predict CRC patients' prognosis. The expression levels of CCL22 (Figure 13(a)), CD36 (Figure 13(b)), CPA3 (Figure 13(c)), CPT1C (Figure 13(d)), KCNE4 (Figure 13(e)), NFATC1 (Figure 13(f)), RASGRP2 (Figure 13(g)), and SLC2A3 (Figure 13(h)) between the high risk score and low risk score were validated based on the 566 CRC samples. Univariate Cox regression analysis results showed that age, KRAS mutation, pathologic T, pathologic N, pathologic M, tumor stage, and risk score were notably associated with CRC patients' prognosis. After multivariate Cox regression analysis, we found that age, KRAS mutation, pathologic M, and risk score could be independent prognostic factors for CRC (Table 4).

### 3.7. The Eight Genes in the Risk Score Are Distinctly Correlated with Molecular Markers of CRC Prognosis

**Figure 14:**
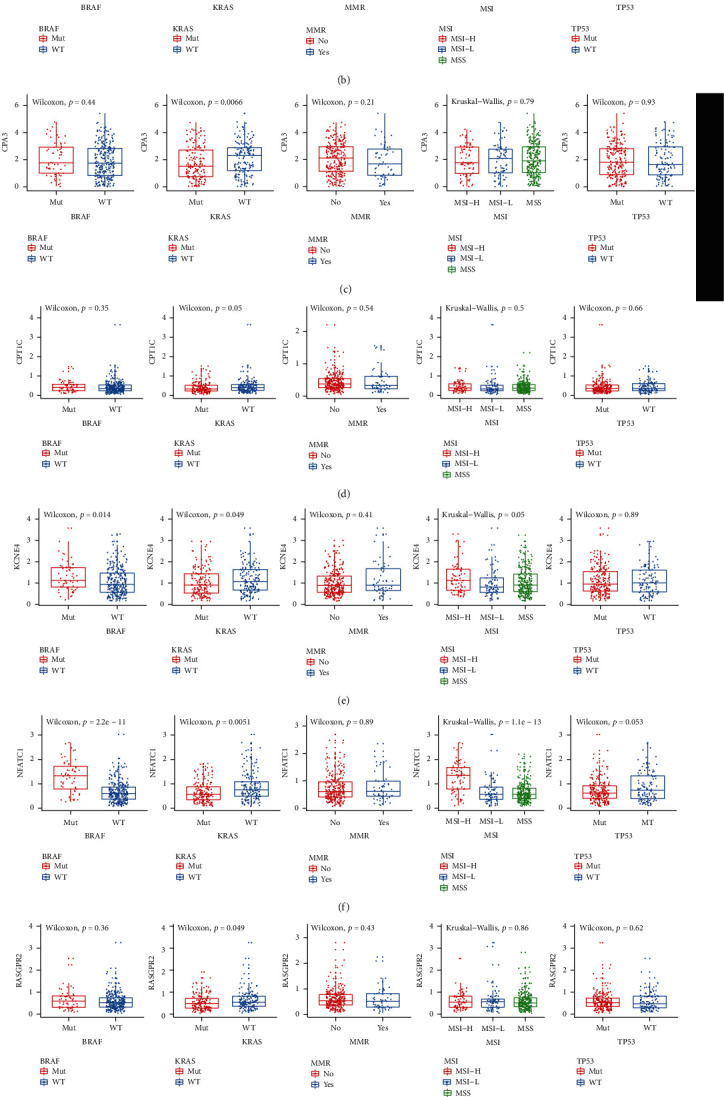
The eight genes in the risk score are distinctly correlated with molecular markers of CRC prognosis including BRAF mutation, KRAS mutation, MMR, MSI, and TP53. (a) CCL22. (b) CD36. (c) CPA3. (d) CPT1C. (e) KCNE4. (f) NFATC1. (g) RASGRP2. (h) SLC2A3.

In Figure 14(a), CCL22 was significantly correlated with BRAF mutation (*p* value = 0.014) and KRAS mutation (*p* value = 0.041). For CD36, there was a distinct correlation between its expression and KRAS mutation (*p* value = 0.00034) and MMR (*p* value = 0.025) in Figure 14(b). CPA3 (*p* value = 0.0066; Figure 14(c)) and CPT1C (*p* value = 0.005; Figure 14(d)) had higher expression levels in KRAS mutation. As shown in Figure 14(e), KCNE4 expression was in significant correlation with BRAF mutation (*p* value = 0.0014), KRAS mutation (*p* value = 0.049), and MSI (*p* value = 0.05). NFATC1 expression was prominently correlated with BRAF mutation (*p* value = 2.2*e*-11), KRAS mutation (*p* value = 0.00051) and MSI (*p* value = 1.1*e*-13) in Figure 14(f). In Figure 14(g), RASGRP2 expression was significantly decreased in KRAS mutation. For SLC2A3, we found that there was a distinct correlation between its expression and BRAF mutation (*p* value = 0.0011) and MSI (*p* value = 0.00013) in Figure 14(h).

## 4. Discussion

In TME, stromal and immune cells are involved in the development of CRC. In this study; using the ESTIMATE algorithm, stromal and immune scores were calculated. A significant correlation between the immune score and CRC patients' prognosis was observed. Both the stromal score and immune score were in significant correlation with clinical characteristics of CRC patients. Furthermore, we identified differentially expressed stromal and immune genes for CRC. Functional enrichment analysis results suggested that these genes were positively related with immune-related pathways like cytokine-cytokine receptor interaction [16, 17] and chemokine signaling pathway [18, 19].

Individual prognosis for CRC patients varies widely. Individual genes often cannot accurately predict the prognosis of patients with CRC. Genes in most prognostic risk scores are screened via differential expression analyses [20–22]. Yet, there are few prognostic models associated with CRC immune infiltration. Therefore, in this study, we selected eight differentially expressed stromal and immune genes related to prognosis for constructing a risk score model. However, focusing only on CRC-related immune-related genes may limit its clinical value. For this reason, through multivariate regression analysis, after adjustment of the clinical characteristics of CRC, we assessed the association between the risk score and CRC prognosis. The results showed that the model may become an independent prognostic factor for CRC. Our risk score exhibited well efficiency in predicting CRC patients' prognosis. Therefore, the risk score model possessed potential prognostic value, which was confirmed using an independent dataset. Among the eight genes in the model, both in the discovery and independent datasets, CCL22 was a protective factor of CRC, while CD36 and NFATC1 were two risk factors of CRC. However, other genes exhibited inconsistent results in the two datasets. This is partly due to the heterogeneity of the samples in the two datasets. Patients in the same pathological stage have different prognosis. Both in the discovery and independent datasets, CCL22 and CPA3 were lowly expressed and KCNE4, NFATC1, and SLC2A3 were highly expressed in the high-risk samples compared to the low-risk samples. However, there were inconsistent results about other genes between the high- and low-risk samples in the two datasets, partly due to the heterogeneity of the samples, different sequencing platforms, different background correction and normalization methods and so on. Thus, it is unreliable to predict CRC patients' prognosis by an individual gene. However, our risk score composed of these genes may accurately suggest the patient's prognosis.

As described in a previous study, high CCL22 expression was found in CRC tissues [23]. Recent study has found that CCL22 secreted by M2 macrophages could mediate CRC 5-FU-mediated chemoresistance [24]. Furthermore, it has been reported that CCL22 was in significant correlation with the infiltrating levels of different T cell subsets for CRC [25]. Our results showed that CD36 was significantly downregulated in CRC tissues compared to normal tissues, which was validated in vitro and in vivo [26]. Genome-wide DNA methylation analysis revealed that hypermethylation of CD36 could contribute to its low expression [27]. Fang et al. found that CD36 expression gradually decreased from adenoma to cancer and CD36 loss implied a poor prognosis in patients with CRC [28]. NFATC1 was deregulated in CRC tissues, which was consistent with previous findings [29]. In vitro, its overexpression significantly promoted CRC cell invasion and metastasis [30]. Kumar et al. reported that NFATC1 indicated poor survival outcomes of CRC patients [31]. High SLC2A3 expression was observed in CRC tissues and its high expression indicated a poor prognosis, consistently with previous research [32, 33]. Furthermore, downregulated CPA3 and RASGRP2 and upregulated CPT1C and KCNE4 were found in CRC tissues, which implied poor prognosis.

As for immune cell infiltration, we found that the eight genes in the risk score model were moderately correlated with the infiltering levels of CD4+T cell, dendritic cell, macrophage, and neutrophil. It has been confirmed that TME affects the efficacy of immunotherapy, and immune cells in TME possess predictive value for immunotherapy treatment [34–36]. Increasing genes have been shown to participate in the regulation of immune cells [37–40]. Therefore, our risk score model could possess potential value to predict CRC patients' prognosis, and the eight genes could become promising immunotherapeutic targets, which deserve further study.

Our correlation analysis results confirmed that the eight genes in the risk score were distinctly correlated with molecular markers of CRC prognosis. However, our study has several limitations. First, our retrospective study limited the application of this risk score. Second, the heterogeneity of the immune microenvironment would inevitably contribute to result bias. Therefore, it is necessary to validate our findings in a prospective clinical study.

## 5. Conclusion

In this study, we conducted an immune-related prognostic model for CRC on the basis of stromal and immune scores. The model had well predictive efficacy for CRC patients' prognosis. Our findings could provide novel biomarkers for predicting the prognosis of CRC patients and developing individualized immunity therapy strategies.

## Figures and Tables

**Table 1:** Clinical characteristics of CRC patients in TCGA datasets (overall = 433).

Characteristics	Groups	*N* (%)
Age (%)	≤60	136 (30.7)
	>60	297 (68.6)

Gender (%)	Female	200 (46.2)
	Male	233 (53.8)

Status (%)	Died	338 (78.1)
	Alive	95 (21.9)

Pathologic T (%)	T1	11 (2.5)
	T2	75 (17.3)
	T3	296 (68.4)
	T4	51 (11.8)

Pathologic N (%)	N0	254 (58.7)
	N1	102 (23.6)
	N2	77 (17.8)

Pathologic M (%)	M0	320 (75.1)
	M1	61 (14.3)
	Mx	45 (10.6)

Tumor stage (%)	I	73 (17.3)
	II	165 (39.1)
	III	123 (29.1)
	IV	61 (14.5)

**Table 2:** Univariate Cox regression analysis results of 23 differentially expressed stromal and immune genes.

Variables	HR	Lower 95% CI	Upper 95% CI	*p* value
CD36	1.381	1.046	1.823	0.02284
KCNE4	1.346	1.004	1.805	0.047303
VEGFC	1.39	1.011	1.912	0.042907
PDE1B	2.242	1.361	3.693	0.001518
BGN	1.166	1.003	1.355	0.046135
CPT1C	2.561	1.476	4.446	8.26E-04
GPX3	1.231	1.03	1.471	0.022285
NGFR	1.344	1.014	1.779	0.039388
SERPINE1	1.173	1.01	1.362	0.037066
CHST1	1.448	1.02	2.055	0.038579
FBLN7	2.648	1.003	6.994	0.049317
KCNJ8	1.372	1.003	1.876	0.047805
SLC2A3	1.225	1.007	1.49	0.041957
CD72	1.714	1.127	2.606	0.011724
APLP1	1.631	1.031	2.58	0.036535
SIGLEC1	1.348	1.019	1.783	0.036725
RASGRP2	1.641	1.044	2.579	0.031925
SPHK1	1.218	1.002	1.481	0.048069
NFATC1	1.674	1.157	2.421	0.006228
LRRN2	1.653	1.139	2.398	0.008126
CCL22	0.686	0.51	0.923	0.012756
CPA3	0.81	0.68	0.966	0.019315
MMP1	0.902	0.816	0.996	0.042396

**Table 3:** Univariate and multivariate Cox regression analyses in a TCGA-CRC cohort.

Characteristics	Univariate analysis		Multivariate analysis	
	HR (95% CI)	*p* value	HR (95% CI)	*p* value
Stromal score	1 (1-1)	0.653	NA	NA
Immune score	1 (1-1)	0.941	NA	NA
Age	1.396 (0.878-2.22)	0.158	NA	NA
Gender	1.127 (0.751-1.692)	0.564	NA	NA
Tumor stage	3.064 (1.986-4.726)	<0.0001	3.320 (0.870-12.640)	0.079
Pathologic T	3.204 (1.398-7.345)	0.006	4.914 (0)	0.996
Pathologic N	2.581 (1.705-3.909)	<0.0001	0.920 (0.290-2.890)	0.880
Pathologic M	3.519 (2.312-5.356)	<0.0001	1.620 (0.84-3.13)	0.151
MMR	0.181 (0.044-0.751)	0.019	0.070 (0.010-0.500)	0.009
BRAF	1.108 (0.620-1.980)	0.729	NA	NA
KRAS	0.912 (0.580-1.434)	0.691	NA	NA
TP53	1.461 (0.884-2.417)	0.139	NA	NA
MSI	0.907 (0.522-1.575)	0.728	NA	NA
Risk score	2.718 (2.063-3.581)	<0.0001	2.420 (1.590-3.700)	<0.0001

NA: not available.

**Table 4:** Univariate and multivariate Cox regression analyses in the GSE39582 dataset.

Characteristics	Univariate analysis		Multivariate analysis	
	HR (95% CI)	*p* value	HR (95% CI)	*p* value
Age	1.455 (1.033-2.051)	0.031	1.590 (1.110-2.260)	0.010
Sex	1.310 (0.980-1.750)	0.068	NA	NA
BRAF mutation	1.111 (0.664-1.861)	0.688	NA	NA
KRAS mutation	1.361 (1.018-1.818)	0.037	1.420 (1.040-1.930)	0.025
TP53 mutation	1.197 (0.844-1.697)	0.312	NA	NA
MMR status	0.768 (0.471-1.252)	0.290	NA	NA
Pathologic T	2.005 (1.025-3.921)	0.042	1.510 (0.770-2.970)	0.229
Pathologic N	1.648 (1.228-2.213)	0.0008	1.090 (0.500-2.380)	0.822
Pathologic M	5.175 (3.621-7.395)	<0.0001	3.880 (2.510-5.990)	<0.0001
Tumor stage	1.767 (1.326-2.354)	0.0001	1.200 (0.520-2.800)	0.670
Risk score	2.718 (2.056-3.595)	<0.0001	2.420 (1.800-3.260)	<0.0001

NA: not available.

## Data Availability

The (data type) data used to support the findings of this study are included within the supplementary information file(s).

## Abbreviations

CRC: Colorectal cancer
TCGA: The Cancer Genome Atlas
ROC: Receiver operating characteristic
OS: Overall survival
PFI: Progression-free survival interval
AUC: Area under the ROC
TME: Tumor microenvironment
FC: Fold change
GO: Gene Ontology
KEGG: Kyoto Encyclopedia of Genes and Genomes
CC: Cellular component
MF: Molecular function
BP: Biological process
PPI: Protein-protein interaction
STRING: The Search Tool for the Retrieval of Interacting Genes.

## Supplementary Materials

Supplementary Materials Supplementary Table 1: the correlation between the eight genes in the risk score model and markers of immune cells.
